# Novel BPI3Vc-vectored chimeric BVDV antigens elicit broadly neutralizing antibodies in cattle

**DOI:** 10.3389/fimmu.2026.1711852

**Published:** 2026-05-18

**Authors:** Huldah Sang, Tae Kim, Rakshith Kumar, Jayden McCall, Aloysius Abraham, Tsvetoslav Koynarski, Michelle Zajac, Karim W. Abdelsalam, Daniela Hernandez Muguiro, Katherine Bauer, Brandon L. Plattner, Waithaka Mwangi

**Affiliations:** 1 Department of Diagnostic Medicine/Pathobiology, Kansas State University, Manhattan, KS, United States; 2 Research Technology Innovation (RTI) Lab, Brookings, SD, United States; 3 Kansas Veterinary Diagnostic Laboratory, Kansas State University, Manhattan, KS, United States

**Keywords:** antigen surface display, Bovine parainfluenza-3 virus, Bovine viral diarrhea virus, cross-protection, efficacy, neutralizing antibody, vaccine, vector

## Abstract

Bovine Viral Diarrhea Virus (BVDV) is a key contributor to the development of Bovine Respiratory Disease complex, which can cause respiratory disease, congenital defects, severe diarrhea, immunosuppression, abortion, or birth of persistently infected calves. Current commercial vaccines are formulated using strains from BVDV-1a and BVDV-2a, conferring protection against some, but not all, homologous species and low neutralization titers against heterologous and emergent species. This study aimed to develop a live vaccine capable of inducing broad protection against diverse BVDV strains. A contemporary live-attenuated BPI3Vc-vectored BVDV prototype vaccine was developed. Novel chimeric BVDV E2-NS2-5^1–2^ antigens, designed based on the consensus of all BVDV-1a, -1b, -2a, -2b, and -2c protein sequences, were used to generate recombinant BPI3Vc_mutant_E2-NS2-5^1–2^ viruses. The recombinant viruses expressed all the ~5kb encoded transgenes, replicated efficiently, remained genetically stable over nine passages *in vitro*, and displayed the E2 ectodomain on the surface of infected cells. Intranasal immunization of calves with a cocktail of these recombinant viruses, designated rBPI3Vc_mut_BVDV, elicited strong serum antibodies against BVDV-1 and -2 viruses compared to a commercial vaccine. Notably, vaccination elicited stronger IgG responses against BVDV-1b CA0401186a and TGAC, with significantly higher virus-neutralizing titers than the commercial vaccine (CA0401186a: p=0.0031; TGAC: p=0.0002). Immunized calves also elicited significantly higher VN titers against BVDV-2a strains 296NC (p=0.0006), 890 (p=0.0020), 296C (p=0.0464), A125 (p=0.0018), and 1373 (p=0.0025). Upon challenge with BVDV-1b CA0401186a, the rBPI3Vc_mut_BVDV vaccinees exhibited a steady weight gain, less decrease in lymphocyte counts, lower viremia, and fewer gross lesions. This data supports use of the live-attenuated BPI3Vc-vector for development of contemporary broadly protective BVDV vaccines that can easily be upgraded for improved disease management and cattle productivity.

## 1 Introduction

Bovine viral diarrhea (BVD) is an infectious disease caused by the Bovine viral diarrhea virus (BVDV), which affects cattle and other ruminants globally (1). The BVDV is a key contributor to the development of Bovine Respiratory Disease complex (BRDC). Infection with the virus can lead to severe diarrhea, respiratory disease, immunosuppression, abortion, congenital defects, and birth of persistently infected (PI) calves, which serve as a major reservoir and shedder of the virus within a herd (2, 3). Immunosuppressed calves are particularly vulnerable to secondary bacterial and fungal infections, which may result in pneumonia or enteritis (4, 5). These secondary infections contribute to high rates of morbidity and mortality in affected animals, resulting in multi-billion dollar losses by the U.S. livestock industry due to BRDC (6).

The pathogen is an enveloped single-stranded RNA virus classified within the family Flaviviridae and genus *Pestivirus* (7). Its genome is approximately 12.5 kb, containing a single open reading frame that encodes for a 450 kDa precursor polyprotein. This polyprotein undergoes proteolytic processing to yield both structural and non-structural proteins. The structural proteins consist of N^pro^, capsid, and glycoproteins E^rns^, E1, and E2, whereas non-structural proteins include the NS2-3, NS4A-B, and NS5A-B (8). The virus is classified into three genotypes, recently updated by the International Committee on Virus Taxonomy as different species (9), based on antigenic differences: BVDV Type 1, comprising 22 subspecies (BVDV-1a to 1v); BVDV Type 2, comprising 4 subspecies (BVDV-2a to 2d); and BVDV Type 3, comprising 4 subspecies (BVDV 3a to 3d) (9–12). Additionally, the virus is categorized into cytopathic or non-cytopathic biotypes, based on the presence or absence of cytopathic effects, respectively, in infected cells (13). *In vivo*, non-cytopathic biotypes are associated with persistent infection, while cytopathic biotypes are linked to mucosal disease (13, 14).

The mainstream BVDV control strategy includes biosecurity practices, diagnosis and elimination of persistently infected animals, and vaccination (15). Vaccination programs primarily utilize Modified Live Virus vaccines (MLVs) or Killed Virus vaccines (KVs), with several commercial vaccines that have been available for decades. The most recognized subspecies in North America were considered to be BVDV-1a, -1b, and -2a (16, 17). Therefore, almost all MLVs or KVs are currently formulated using a combination of one representative strain from BVDV-1a and one from BVDV-2a (18, 19). Interestingly, BVDV-1b is now the most predominant species in the United States (16, 17, 20, 21). Additionally, BVDV 2b and 2c were recently identified, with genetic and antigenic differences from BVDV-2a (22). Notably, studies indicate that sera from cattle immunized with various BVDV-2a MLV vaccines varied in ability to neutralize BVDV-2b and 2c strains. Specifically, this study reported that the vaccine strains elicit protective neutralization titers against some, but not all, BVDV-1a, -1b, and -2a strains and significantly low neutralizing titers against BVDV-2c strains (18). Similar outcomes have been observed in other studies, where species-specific MLV vaccines were ineffective at conferring cross-protection against diverse BVDV strains, in Brazilian (23, 24) and European livestock (25, 26). This strongly suggests that there are significant antigenic differences between some commercial vaccines and circulating field strains. Current MLV vaccines require updating, as they lack broad epitope coverage across diverse circulating BVDV strains and emerging variants. Additionally, as each BVDV strain has both shared and strain-specific neutralizing epitopes, a vaccine that can capture this diverse epitope repertoire is expected to confer broad protection.

Colostrum-derived BVDV-specific neutralizing antibodies provide the initial control against BVDV infection in neonates and yearlings (27). However, as the neutralizing antibody titers decay, calves become vulnerable to infection at different time points, and in the presence of persistently infected (PI) calves in the herd, there is always a high risk of infection (28). Current MLVs and KVs are administered parenterally (19, 26, 29–31), with the MLVs having the advantage of replicating *in vivo* thereby mimicking wild-type virus infection. However, in calves less than six months old, parenterally administered vaccines are highly susceptible to maternal antibody neutralization and complement-mediated clearance that limits their efficacy (32–34). Consequently, immunization with BVDV-1 and -2 vaccines is usually delayed for about six months to allow maternal antibodies to wane. Unfortunately, in the presence of PI calves in the herd, there is always a high risk of infection as antibodies can decay below protective titers earlier than six months (27, 28, 35). Additionally, some calves entering feedlots are PI, and the lack of effective chute-side testing to identify them in a timely manner poses a great risk to unprotected cattle (16). Therefore, improved vaccines are needed to ensure a more consistent control of BVDV infection, especially in calves less than six months old.

Rationally designed live-vectored BVDV subunit vaccines delivered intranasally offer an attractive alternative, as the replicating vector targets the respiratory tract, the primary portal of BVDV entry, thereby priming virus-specific immune responses in the presence of circulating neutralizing maternal antibodies. Protective BVDV antigens have been identified. The E2 and NS2–3 antigens are immunodominant, inducing neutralizing antibodies and T cell responses that confer protection against wild-type virus (36–39). Conserved regions within these antigens also contain MHC *DR*-restricted T cell epitopes (35, 37, 40–42). Additionally, the NS4–5 antigens contain CD4^+^ T-cell epitopes that induce strong IFN-γ secretion (40, 43), and highly conserved CD8^+^ T cell epitopes capable of potent IFN-γ-induction across diverse BVDV strains (38). Although conserved regions exist within E2, NS2-3, and NS4-5 (44), these antigens, particularly E2, also contain variable regions that are crucial for eliciting protective neutralizing antibodies, thus contributing to the variable cross-reactivity observed among BVDV strains which, in part, explains why current vaccines are poor at conferring cross-protection against diverse BVDV strains (23). A vaccine containing all defined protective B and T cell epitopes from diverse BVDV-1 and -2 strains is yet to be developed and tested in calves. We hypothesized that a rationally designed prototype vaccine containing consensus E2, NS2-3, and NS4–5 antigens, which incorporates protective epitopes conserved among BVDV-1a, 1b, and BVDV-2 (a, b, c) subspecies as well as strain-specific protective epitopes, would confer broad protection against diverse BVDV-1 and -2 strains. This approach also enables rapid vaccine updates by incorporating protective determinants from emerging variants.

In this study, a previously described live attenuated Bovine parainfluenza 3 virus genotype c vector, designated BPI3Vc_mut_ (45), was used for intranasal delivery of chimeric BVDV E2-NS2-5^1–2^ antigens, derived from the consensus of more than 200 sequenced BVDV-1 and -2 antigenic determinants in the United States as of 2019. The E2 structural glycoproteins were designed for multicistronic expression and surface display on infected cells for optimal recognition by B-cells. A cocktail of the recombinant BPI3Vc_mut-_vectored BVDV antigens, designated rBPI3Vc_mut_BVDV, was evaluated for its multicistronic antigen expression, surface display of the E2 structural antigens, immunogenicity, and protective efficacy in five-month-old Holstein calves following challenge.

## 2 Materials and methods

### 2.1 Cells and viruses

Baby Hamster Kidney cells constitutively expressing T7 RNA polymerase (BSR T7/5 cells), Human Embryonic Kidney (HEK) 293A cells and Madin–Darby bovine kidney (MDBK) cells (Millipore Sigma, MDBK NBL-1) were maintained as previously described (45). Wild-type BVDV-1b (strains CA0401186a, and TGAC) and BVDV-2a (strains A125 and 1373) viruses were obtained from RTI Labs (Brookings, SD). The BVDV viruses were propagated and titrated in 80% confluent MDBK cells at 37 °C with 5% CO_2_.

### 2.2 Monoclonal and polyclonal antibodies

Mouse anti-Flag tag monoclonal antibody (mAb) clone M2 and mouse anti-His tag mAb clone HIS.H8 were sourced from Millipore Sigma (F1804) and Invitrogen (MA1-21315), respectively. The neutralizing mouse mAb against BVDV-1 and -2 E2 glycoprotein was sourced from VMRD (Cat # 348, Pullman WA). Polyclonal IgG antibodies (pAbs) 2539 against BVDV-1, which also cross-reacts with BVDV-2, were purified by protein-G affinity chromatography from sera of a steer hyperimmunized with Gamma irradiated BVDV-1b TGAC (38). The AR22.7 mouse mAb against *Theileria parva* modified surface protein was obtained from Washington State University (46). The mAbs were used at 4 µg/ml, whereas the purified IgGs were used at 6 µg/ml for immunofluorescence and immunocytometric analyses, respectively. For Western blots, the mAbs were used at 1.5 µg/ml.

### 2.3 Design and generation of plasmid constructs

The pFLC-BPI3Vc_mut_ plasmid construct containing the BPI3Vc_mut_ backbone was previously described (45). Five novel chimeric BVDV antigen expression cassettes were designed: E2^1a^-2A-NS2-3^1a^; E2^1b^-2A-NS2-3^1b^; and E2^2^-2A-NS2-3^2^, encoding consensus chimeric E2-NS2–3 polypeptides derived from the complete E2 and NS2–3 sequences of BVDV-1a, -1b, and BVDV-2, respectively. An autocleavable 2A motif was inserted downstream of E2 to enable independent co-expression. NS4–5^1^ and NS4-5^2^, encoding consensus sequences from BVDV-1 and BVDV-2 genomes, were also designed. Genes encoding the E2^1a^-2A-NS2-3^1a^; E2^1b^-2A-NS2-3^1b^; and E2^2^-2A-NS2–3^2^ cassettes, designated E2-NS2-3^1a,1b,2^, and the NS4–5^1^ and NS4–5^2^ cassettes, designated NS4-5^1,2^, were codon-optimized and synthesized (GenScript). These cassettes, collectively designated E2-NS2-5^1-2^, were cloned into the pFLC-BPI3Vc_mut_ plasmid. The resultant constructs were validated by DNA sequencing and used to rescue respective recombinant viruses that were tested for protein expression as previously described (45).

Briefly, all available E2^1a^-NS2-3^1a^, E2^1b^-NS2-3^1b^, E2^2^-NS2-3^2^, NS4-5^1^, and NS4–5^2^ protein sequences from North American BVDV strains as of 2019 were retrieved from NCBI, including 41 BVDV-1a, 51 BVDV-1b, and 112 BVDV-2 E2-NS2–3 sequences, along with 77 BVDV-1 and 101 BVDV-2 NS4–5 protein sequences. Multiple sequence alignment performed using MEGA 7 generated consensus amino acid sequences based on a >50% identity threshold. When sequence identity was below 50%, the dominant amino acid at each position was selected. The final consensus sequences for the chimeric E2-NS2-3^1b^ and NS4–5^2^ polypeptides were selected to generate maximum likelihood phylogenetic trees. The resulting consensus FASTA files were analyzed to assess the conservation of chimeric E2-NS2-5^1–2^ sequences relative to their respective parental proteins.

The E2^1a^, E2^1b^, E2^2^ and NS2-3^1a^, NS2-3^1b^, NS2–3^2^ protein sequences were designed as multicistronic expression cassettes, separated by a 22 amino acid ‘P2A’ autocleavable motif (GSGATNFSLLKQAGDVEENPGP) derived from porcine teschovirus-1 (P2A) (47), while the NS4-5^1^, NS4–5^2^ protein sequences were designed as monocistronic expression cassettes. In addition, a gene encoding an irrelevant antigen, *Theileria parva* modified surface protein (TMSP) (46), was also designed as a monocistronic expression cassette. To enable antigen display on the surface of infected cells, sequences encoding the transmembrane and cytoplasmic domains of BPI3Vc Fusion protein were added, in-frame, downstream of the E2^1a^, E2^1b^, E2^2^, and TMSP protein sequences. A Flag tag sequence (DYKDDDDK) was added in-frame, at the N-terminal, and a His tag sequence (HHHHHH) at the C-terminal, of each of the chimeric E2^1a^-2A-NS2-3^1a^ (~5.2 kb), E2^1b^-2A-NS2-3^1b^ (~5.2 kb), E2^2^-2A-NS2-3^2^ (~5.2 kb), NS4-5^1^ (~5 kb), NS4-5^2^ (~5 kb), and the TMSP (~2.6 kb) protein sequences. The resulting polypeptide sequences were used to generate synthetic genes codon optimized for *Bos taurus* expression and cloned into pcDNA 3.1 (+) mammalian expression vector (GenScript) to generate constructs designated pcDNA3.1-E2^1a^-2A-NS2-3^1a^, pcDNA3.1-E2^1b^-2A-NS2-3^1b^, pcDNA3.1-E2^2^-2A-NS2-3^2^, pcDNA3.1-NS4-5^1^, and pcDNA3.1-NS4-5^2^. Protein expression by these constructs was evaluated by transfecting HEK293A cells and immunostaining at 48 hours post-transfection using anti-Flag mAb-AP, anti-His mAb, mAb 348 or pAb 2539, followed by an anti-mouse or anti-bovine IgG-AP-labeled secondary antibodies, respectively (Jackson ImmunoResearch, 115-055-003, 101-055-003).

**Figure 1:**
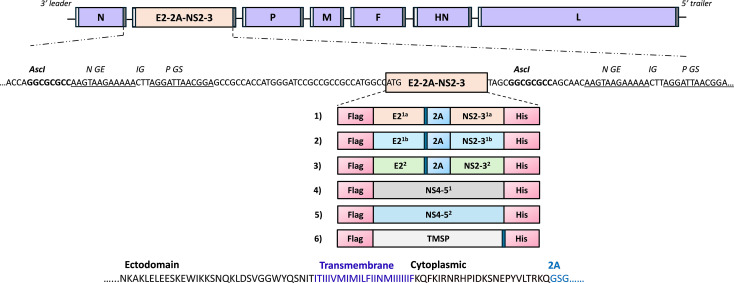
Attenuated BPI3Vc expression constructs encoding BVDV antigens. Design of the BPI3Vc constructs encoding chimeric BVDV 1a, 1b, and 2 antigenic cassettes: 1) E2-2A-NS2-3^1a^; 2) E2-2A-NS2-3^1b^; 3) E2-2A-NS2-3^2^; 4) NS4-5^1^; and 5) NS4-5^2^. 6) TMSP represents a negative control antigen cassette. Transgenes are flanked by gene start (white bar) and gene end (gray bar) transcription signals, and cloned, in-frame, between the genes encoding N and P. All genes are separated by an intergenic trinucleotide sequence (CTT), for expression as separate mRNA. E2-NS2-3^1a,1b,2^ antigenic cassettes were designed for multicistronic expression separated by an auto-cleavable domain, 2A. The E2^1a,1b,2^ and TMSP cassettes contain transmembrane and cytoplasmic domains of the BPI3Vc fusion protein. Flag tag and His tag genes are added in-frame at the N’ end and C’ end, respectively, to assess recombinant protein expression.

Genes from the pcDNA3.1-E2^1a^-2A-NS2-3^1a^, pcDNA3.1-E2^1b^-2A-NS2-3^1b^, pcDNA3.1-E2^2^-2A-NS2-3^2^, pcDNA3.1-NS4-5^1^, and pcDNA3.1-NS4–5^2^ subclones with the best protein expression along with a previously validated pcDNA3.1-TMSP construct (GenScript), were used as templates for PCR amplification and subcloning of the transgenes into the pFLC-BPI3Vc_mut_ plasmid construct to generate recombinant plasmid constructs, designated pFLC-BPI3Vc_mut_E2^1a^-2A-NS2-3^1a^, pFLC-BPI3Vc_mut_E2^1b^-2A-NS2-3^1b^, pFLC-BPI3Vc_mut_E2^2^-2A-NS2-3^2^, pFLC-BPI3Vc_mut_NS4-5^1^, pFLC-BPI3Vc_mut_NS4–5^2^ and pFLC-BPI3Vc_mut_TMSP, respectively, which were used for virus assembly. Briefly, genes encoding the chimeric E2^1a^-2A-NS2-3^1a^, E2^1b^-2A-NS2-3^1b^, E2^2^-2A-NS2-3^2^, NS4-5^1^, NS4-5^2^, and TMSP were PCR-amplified using the primers shown in **Table 1**. The primers introduced an AscI restriction site, along with essential elements for cloning transgenes, between the N and P genes of the backbone (**Figure 1**), while maintaining the ‘rule of six’ (49), to ensure efficient assembly of the recombinant virus and replication of the progenies, as previously described (45). The resulting PCR products were cloned into the pFLC-BPI3Vc_mut_ backbone via the AscI site (**Figure 1**), as previously described (45), and positive constructs were identified by PCR colony screening using a vector-specific forward primer and gene-specific reverse primers (**Table 1**). AscI restriction digest of positive pFLC-BPI3Vc_mut_E2^1a^-2A-NS2-3^1a^, pFLC-BPI3Vc_mut_E2^1b^-2A-NS2-3^1b^, pFLC-BPI3Vc_mut_E2^2^-2A-NS2-3^2^, pFLC-BPI3Vc_mut_NS4-5^1^, pFLC-BPI3Vc_mut_NS4-5^2^, and pFLC-BPI3Vc_mut_TMSP plasmid minipreps was used to confirm presence of ~5 kb BVDV transgenes and ~2.6 kb TMSP transgene, respectively. DNA sequencing was used to validate gene integrity using primers shown in **Table 1**.

**Table 1:** Primers used to amplify genes of interest.

Gene	Primer name	Sequence (5’ → 3’)
E2-NS2-3^1,2,3^	Forward-1	GTAAGAAAAACTTAGGATTAACGGAGCCGCCACCATGGGATCCACGGCCGCCGCCGCCATGGCCATCGCGTATGATCACCATAGTTG
	Forward-2	GATTCTCGTATCGTATCT**GGCGCGCC**AAGTAAGAAAAACTTAGGATTAACGGAGCCGC
	Reverse	CGTGATAGT**GGCGCGCC**GCTAGCTATAGCGGCCGCATCATTACTAGTGGTGATGGTGATG
Chimeric NS4-5^1^	Forward-1	GAAAAACTTAGGATTAACGGAGCCGCCACCATGGGATCCACGCGTGCCGCCGCCGCCATGGCCATGGACTACAAGGACGATGACGATAAG
	Forward-2	GATTCTCGTATCGTATCT**GGCGCGCC**AAGTAAGAAAAACTTAGGATTAACGGAGCCGC
	Reverse	CGTGATAGT**GGCGCGCC**GCTAGCTATAGCGGCCGCATCATTACTAGTGGTGATGGTGATG
Chimeric NS4-5^2^	Forward-1	GAAAAACTTAGGATTAACGGAGCCGCCACCATGGGATCCACGCGTGCCGCCGCCGCCATGGCCATGGACTACAAGGACGATGACGATAAG
	Forward-2	GATTCTCGTATCGTATCT**GGCGCGCC**AAGTAAGAAAAACTTAGGATTAACGGAGCCGC
	Reverse	CGTACT**GGCGCGCC**GCTAGCTATAGCGGCCGCATTATCATTACTAGTGGTGATGGTGATG
TMSP	Forward-1	ACTGTGATAATAGTACGCGTGCCGCCGCCGCCATGGCCATGGCCATGACCACCATAGTTG
	Forward-2	TAAGAAAAACTTAGGATTAACGGAGCCGCCACCATGGGATCCACGCGTGCCGCCGCCGC
	Forward-3	GATTCTCGTATCGTATCT**GGCGCGCC**AAGTAAGAAAAACTTAGGATTAACGGAGCCGC
	Reverse-1	AGCTGTAGTGATAATAGTGCGGCCGCATTATCATTACTAGTGGTGATGGTGATGATG
	Reverse-2	CGTACT**GGCGCGCC**GCTAGCTATAGCGGCCGCATTATCATTACTAGTGGTGATGGTGATG
Colony Screening	Vector Forward	AGGGCAGCCTGAATCCAGAGGAGATCAGGATCA
	E2-NS2-3^1,2^ Reverse	TATGGACGATGACGATAAGTCCCTCGAGGCCAGTCCCACCACCTGCTTCAG
	E2-NS2–3^3^ Reverse	TATGGACGATGACGATAAGTCCCTCGAGGCCAGGCCGGTCACCTGCTTCAG
	NS4–5^1^ Reverse	CGTGATAGT**GGCGCGCC**GCTAGCTATAGCGGCCGCATCATTACTAGTGGTGATGGTGATG
	NS4–5^2^ Reverse	CGTACT**GGCGCGCC**GCTAGCTATAGCGGCCGCATTATCATTACTAGTGGTGATGGTGATG
	TMSP Reverse	AGCTGTAGTGATAATAGTGCGGCCGCATTATCATTACTAGTGGTGATGGTGATGATG

Bold text denotes the AscI restriction site.

### 2.4 Generation and validation of recombinant viruses

Recombinant viruses were assembled by co-transfecting BSRT T7/5 cells with 3 µg of the antigenome plasmid constructs (pFLC-BPI3Vc_mut_E2^1a^-2A-NS2-3^1a^, pFLC-BPI3Vc_mut_E2^1b^-2A-NS2-3^1b^, pFLC-BPI3Vc_mut_E2^2^-2A-NS2-3^2^, pFLC-BPI3Vc_mut_NS4-5^1^, pFLC-BPI3Vc_mut_NS4-5^2^, or pFLC-BPI3Vc_mut_TMSP) along with 1.6 µg pcDNA3.4-N, 1.6 µg pcDNA3.4-P, and 0.8 µg pcDNA3.4-L helper plasmids (45). Recombinant viruses were rescued by co-culture of the transfected BSRT T7/5 cells with MDBK cells as previously described (45). The recombinant viruses generated were designated rBPI3Vc_mut_E2^1a^- NS2-3^1a^, rBPI3Vc_mut_E2^1b^-NS2-3^1b^, rBPI3Vc_mut_E2^2^-NS2-3^2^, rBPI3Vc_mut_NS4-5^1^, rBPI3Vc_mut_NS4-5^2^, and rBPI3Vc_mut_TMSP, respectively. Cell pellets and supernatants were processed to generate seed viral stocks, which were snap-frozen at -80 °C. A ten-fold diluted stock virus was used to validate protein expression by immunostaining of infected MDBK cells or immunofluorescence assay (IFA) as previously described (45). Protein expression by the rBPI3Vc_mut_E2^1a^-NS2-3^1a^, rBPI3Vc_mut_E2^1b^-NS2-3^1b^, rBPI3Vc_mut_E2^2^-NS2-3^2^, rBPI3Vc_mut_NS4-5^1^, rBPI3Vc_mut_NS4–5^2^ or the rBPI3Vc_mut_TMSP viruses was validated using the anti-Flag mAb-AP, anti-His mAb, the mAb 348 against BVDV-1 and -2 E2 glycoprotein, the bovine pAb 2539 against BVDV, or the mAb AR22.7 against TMSP. Secondary antibodies used for IFA, including a goat anti-mouse IgG-FITC (Jackson ImmunoResearch) and a goat anti-bovine IgG-FITC (Jackson ImmunoResearch), were diluted at 1:1000. Uninfected wells were included as negative controls. Images of the cells expressing the target proteins were captured using an AmScope MU1803-HS digital camera, and the best-expressing clone for each construct was selected for virus expansion in MDBK cells followed by virus purification over a 30-70% sucrose gradient, as previously described (45).

Incorporation into the BPI3V virions of the individual ~ 53 kD E2^1a^, E2^1b^, E2^2^, ~ 135 kD NS2-3^1a^, NS2-3^1b^, NS2–3^2^ antigens by the multicistronic cassettes, or the ~ 185 kD NS4-5^1^, NS4–5^2^ and ~87 kD TMSP antigens by the monocistronic cassettes, was confirmed by Western Blot analysis of purified viruses, as previously described (45), using 1.5 µg/ml mAb 348 followed by a goat anti-mouse IgG-HRP diluted at 1:1000 (Jackson ImmunoResearch), 1.5 µg/ml of anti-Flag mAb-HRP (Sigma Aldrich), or 1.5 µg/ml of mouse anti-His mAb (Invitrogen) followed by a goat anti-mouse IgG-HRP diluted at 1:1000 (Jackson ImmunoResearch).

### 2.5 Flow cytometric analysis

The E2^1a^, E2^1b^, E2^2^ and TMSP polypeptides, designed with the transmembrane and cytoplasmic domains of BPI3Vc Fusion protein to anchor the E2 and TMSP ectodomains on the surface of infected cells, were evaluated for surface display by flow cytometry as previously described (45). Briefly, 80% confluent 6-well MDBK cells were infected with the rBPI3Vc_mut_E2^1a^-NS2-3^1a^, rBPI3Vc_mut_E2^1b^-NS2-3^1b^, rBPI3Vc_mut_E2^2^-NS2-3^2^, rBPI3Vc_mut_TMSP, or an irrelevant rBPI3Vc_mut_COV19 Spike virus at an MOI of 1. The infected cells were incubated at 37 °C for 2 days, then harvested and processed for analysis, as previously described (45). Surface staining was performed using mAb 348 against E2^1a^, E2^1b^, E2^2^ (1:250 dilution) or mAb AR22.7 against TMSP (1:250 dilution), followed by a goat anti-mouse IgG-FITC (1:250 dilution). Data was acquired and analyzed as previously described (45).

### 2.6 Viral growth curves

Multicycle replication growth curves of representative recombinant viruses expressing BVDV-1 and -2 antigens, including negative control viruses, were performed as previously described (45). Briefly, 80% confluent 6-well MDBK cells were infected at an MOI of 1 in duplicates with the following viruses: i) rBPI3Vc_mut_E2^1a^-NS2-3^1a^; ii) rBPI3Vc_mut_NS4-5^2^; iii) rBPI3Vc_mut_TMSP; or iv) wild-type BPI3Vc TVMDL16 virus. The infected cells were incubated at 37 °C for four days. Cultures were gently mixed every 24 hours, and a 0.5 ml sample was aliquoted and replenished with an equal volume of fresh media (duplicate aliquots were pooled for virus titration). The virus aliquots were snap-frozen at -80 °C, and virus titers from each time point were determined by TCID_50_ calculation as previously described (45).

### 2.7 Viral RNA extraction and sequencing

MDBK cells grown in 6-well plates were infected at 80% confluence with representative Passage 1 (P1) viruses expressing BVDV-1 and -2 antigens, including rBPI3Vc_mut_E2^1a^-NS2-3^1a^, rBPI3Vc_mut_E2^1b^-NS2-3^1b^ and rBPI3Vc_mut_NS4-5^2^, at an MOI of 1, and incubated at 37 °C for four days. The resulting P2 virus was diluted ten-fold and infected onto a fresh MDBK monolayer. After nine passages, cell pellets from the P2 and P9 viruses were resuspended in 1ml Trizol (Thermo Fisher Scientific, 15596026), and RNA was extracted and sequenced using the Illumina platform as previously described (45). The percentage of sequence identity of the P9 progeny viruses was compared to P2 progenies and the original plasmid sequences.

### 2.8 Immunization

Fifteen five-month-old male Holstein calves (obtained from J.R. Livestock Inc, Iowa), seronegative for BPI3V, BVDV-1 and -2 as confirmed by sera neutralization tests (performed by Iowa State University Veterinary Diagnostic Laboratory), were included in this study. The calves were randomly allocated to three groups (n=5) (**Table 2**). Seven days after acclimatization, each calf in Group 1 was immunized intranasally with a cocktail of the rBPI3Vc_mut_E2^1a^-NS2-3^1a^, rBPI3Vc_mut_E2^1b^-NS2-3^1b^, rBPI3Vc_mut_E2^2^-NS2-3^2^, rBPI3Vc_mut_NS4-5^1^, rBPI3Vc_mut_NS4–5^2^ virus constructs, designated rBPI3Vc_mut_BVDV (4.4 x 10^9^ TCID_50_ in 2ml), followed by a first and second booster dose of 4.4 x 10^9^ TCID_50_ in 2ml and 8.4 x 10^10^ TCID_50_ in 2ml, administered on weeks 3 and 6 post-priming, respectively. All intranasal immunizations were conducted using an intranasal mucosal atomization device (Teleflex MAD 300). Each calf in Group 2 received a subcutaneous injection of the recommended 2ml commercial Bovi-Shield Gold 5 vaccine (Zoetis) for the prime and booster doses. The commercial vaccine is a multivalent Modified Live Virus (MLV) vaccine against Infectious Bovine Rhinotracheitis, Bovine Viral Diarrhea Virus 1 and 2, Parainfluenza 3 virus, and Bovine Respiratory Syncytial Virus. Calves in Group 3 served as negative controls and were immunized intranasally with the irrelevant rBPI3Vc_mut_TMSP virus at 1.4 x 10^9^ TCID_50_ in 2ml for the priming dose, followed by a first and second booster dose of 2.9 x 10^9^ TCID_50_ in 2ml and 1.2 x 10^10^ TCID_50_ in 2ml, respectively. All priming and booster viruses were back-titrated after administration. The three groups of calves were housed separately during the immunization phase. Their rectal temperature and body weights were recorded weekly.

**Table 2:** BVDV calf immunization protocol.

Group	ID	Immunogen	Route	Prime-boost dose/calf
**1. rBPI3Vc_mut_BVDV**	5673	*Recombinant BPI3Vc_mut_BVDV cocktail viruses:* rBPI3Vc_mut_BVDV E2-NS2-3^1a^; E2-NS2-3^1b^; E2-NS2-3^2^; NS4-5^1^; and NS4–5^2^ viruses	IN	4.4 x10^9^ TCID_50_, 4.4 x10^9^ TCID_50_, 8.4 x10^10^ TCID_50_
	5694			
	5715			
	5717			
	5725			
**2. Bovi-Shield Gold 5** (Zoetis)	5656	*Commercial MLV cocktail:* Infectious bovine rhinotracheitis Bovine viral diarrhea virus 1 and 2 Parainfluenza 3 virus Bovine respiratory syncytial virus	SQ	2 ml, 2 ml, 2ml
	5657			
	5688			
	5698			
	5723			
**3. rBPI3Vc_mut_TMSP**	5663	Recombinant BPI3Vc_mut_TMSP virus	IN	1.4 x10^9^ TCID_50_; 2.9 x 10^9^ TCID_50_; 1.2 x10^10^ TCID_50_
	5696			
	5710			
	5719			
	5724			

Bold text denotes the type of vaccine per group.

### 2.9 Evaluation of antibody responses

Whole blood was collected every week, and sera were assayed for recognition of BVDV wild-type viruses by IFA, then antibody titers were determined by using serial sera dilutions as previously described (48). Briefly, whole blood collected in coagulation tubes was centrifuged at 21 °C and 3500 rpm for 15 minutes to obtain sera. MDBK cells grown in 6-well plates were infected at 80% confluency in duplicates with the BVDV-1b strains (CA0401186a, TGAC) or BVDV-2a strains (A125 and 1373) at an MOI of 1. Uninfected wells were included as negative controls. After incubation for three days at 37 °C, the plates were fixed with cold methanol for 5 minutes, blocked with 5% FBS in PBS for 1 hr. at 37 °C, and probed with sera collected one week after the second boost, diluted at 1:100, followed by a goat anti-bovine IgG-FITC (1:1,000 dilution, Jackson ImmunoResearch). Plates were evaluated using an Olympus CKX53 fluorescent microscope, and images were captured using an AmScope MU1803-HS digital camera. To determine the endpoint titer, MDBK cells were grown in 96-well plates to 80% confluency and infected with the BVDV-1b strains (CA0401186a, TGAC) or BVDV-2a strains (A125, 1373) as described above for three days at 37 °C. Plates were fixed and probed with sera from blood collected from the calves one week after the second boost as described above. Sera were serially diluted two-fold from 1:250 to 1:4000 and used to probe the infected cells in duplicates. Antibody titers are expressed as the reciprocal of the highest serum dilution where staining was observed.

### 2.10 Virus neutralization

Neutralization of wild-type BVDV-1a (Singer, NADL), BVDV-1b (CA0401186a, TGAC), or BVDV-2a (1373, 296NC, 890, 296C, A125) viruses was evaluated using sera from blood collected one week after the second boost as previously described (45). The presence or absence of virus in the cytopathic BVDV-1 (Singer, NADL, and TGAC) and BVDV-2 (A125, and 296C) was confirmed by observing CPE. Presence of virus in the non-cytopathic BVDV-1 (BJ, and CA0401186a) and BVDV-2 (890,1373, and 296NC) was confirmed by immunoperoxidase assay (49). Virus neutralization titers were expressed as the reciprocal of the highest dilution in which more than 50% CPE or staining for the virus was detected.

### 2.11 Animal challenge

Sixty days after priming, all the calves were challenged by intranasal instillation of 3.4 x 10^5^ TCID_50_ of BVDV Type 1b, CA0401186a virus (50), with the challenge virus back-titrated after administration. BVDV-1b is the most prevalent strain in North America and is recommended for conducting BVDV vaccine efficacy studies (51). The calves were monitored daily for 18 days for clinical signs, including changes in breathing, liveliness, ocular and nasal discharges, coughing, changes in appetite, diarrhea, and any indications of depression. Rectal temperatures were recorded daily, and body weights were measured before challenge, six- and twelve-days after challenge. Uncoagulated blood was collected every three days. After 18 days, all animals were humanely euthanized for necropsy following approved protocols at Kansas Veterinary Diagnostic Laboratory.

### 2.12 Hematology and viremia

On days 0, 3, 6, 9, 12, 15, and 18 post-challenge, uncoagulated blood was drawn into K2 EDTA-coated tubes to evaluate white blood cell counts, lymphocyte counts, and viremia. Automated hematology analysis was performed at Kansas Veterinary Diagnostic Laboratory, as previously described (45). Hematologic parameters, including white blood cell (WBC) and lymphocyte (LYMPH) counts, were recorded and verified by a staff member and a veterinary clinical pathologist (DHM) via blood smear examination. To establish a common baseline, the mean WBC or lymphocyte count for each group on DPC 3, 6, 9, 12, 15, and 18 were subtracted from their respective initial values on DPC 0, and the resulting differences were plotted as mean count ratios relative to baseline.

Titers of BVDV-1b CA0401186a wild-type virus in blood after challenge were determined by titrating samples in MDBK cells followed by IFA, as described in previous studies (39, 50, 52). Briefly, blood samples underwent three freeze-thaw cycles, centrifuged, and the resulting lysates were diluted ten-fold in DMEM. 96-well MDBK cell monolayers were then infected with 50 µl of each serially diluted lysate, with nine replicate wells per sample. After incubation at 37 °C for five days, the plates were fixed in formalin and stained using the previously mentioned mAb 348 and a goat-anti mouse IgG-FITC (Jackson ImmunoResearch). The BVDV titer was determined as the highest dilution at which positive staining was observed.

### 2.13 Necropsy and pathology

Three weeks after challenge, the study was terminated and all calves were sedated and euthanized by penetrative captive bolt. Complete necropsy examination, analysis of gross lung lesions, and histopathologic assessment of the cranial, middle, and caudal lobes of the right lung and the cranial and caudal lobes of the left lung were directed by board-certified anatomic pathologists who were blinded to the animal groupings, at Kansas Veterinary Diagnostic Laboratory, as previously described (45). Morphologic diagnosis of histologic lesions was graded as mild, moderate, or severe and assigned scores reflecting the presence and severity of each lesion as (0= normal; 1 = lesion is present; 2 = mild; 3 = moderate; 4 = severe).

### 2.14 Statistical analysis

GraphPad Prism10 was used to analyze data. The significance of differences in mean antibody titer between the three treatment groups during the different sampling time points was analyzed by Two-way repeated measures ANOVA followed by Tukey correction for multiple mean comparisons. For the blood viremia data analysis, the significance of differences between the treatment and control groups was analyzed by One-Way ANOVA followed by Dunnett’s T3 correction for multiple comparisons. Statistical differences with significance levels of p < 0.05 was considered significant.

## 3 Results

### 3.1 Recombinant viruses expressed chimeric BVDV antigens

**Figure 2:**
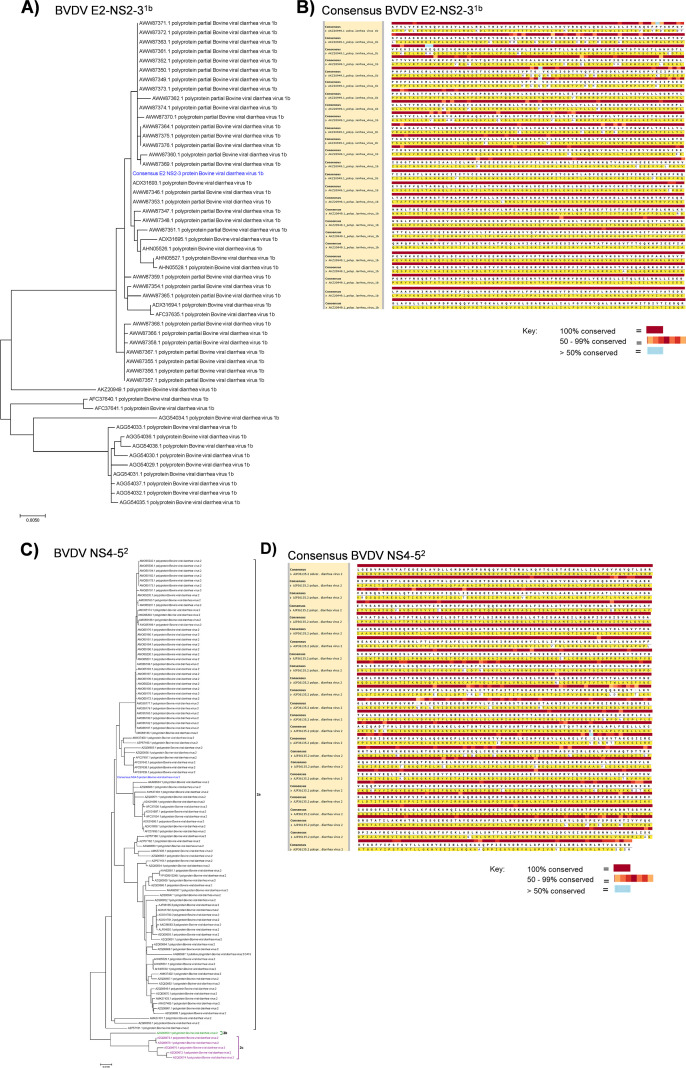
Design of chimeric BVDV antigens. Maximum likelihood phylogenetic tree showing clustering of the consensus E2-NS2-3^1b^ sequence **(A)** and NS4–5^2^ sequence **(C)** (shown in blue) derived from 51 BVDV E2-NS2-3^1b^ and 101 BVDV NS4–5^2^ polypeptide sequences, respectively. Level of conservation of chimeric E2-NS2-3^1b^ **(B)** and NS4-5^2^ **(D)** polypeptide sequences, respectively.

A consensus of 44, 51, and 112 genomes for BVDV-1a, BVDV-1b, and BVDV-2 generated the novel chimeric E2-NS2-3^1a^, E2-NS2-3^1b^, and E2-NS2–3^2^ polypeptide sequences, respectively, whereas a consensus of 77 and 101 genomes for BVDV-1 and BVDV-2 generated the novel chimeric NS4–5^1^ and NS4–5^2^ polypeptide sequences, respectively (**Figures 2A, C**). The chimeric sequences exhibited varying levels of identity with their corresponding parental protein sequences. Specifically, the chimeric E2¹^b^; and NS2–3¹^b^ sequences exhibited 75.6% and 93.2% identity, respectively, among the 51 E2 and NS2–3 BVDV-1b protein sequences analyzed (**Figure 2B**). The chimeric NS4–5² sequence showed 70.7% identity with the 101 NS4–5 BVDV-2 protein sequences analyzed (**Figure 2D**). Additionally, the chimeric E2¹^a^ and NS2–3¹^a^, E2² and NS2–3², and NS4–5² polypeptides showed 36.3% and 77.9%, 57.3% and 75.6%, and 72.8% identity, respectively, with their corresponding parental protein sequences. This indicates that the E2 glycoprotein was the most heterogeneous antigen. To evaluate the expression and antigenicity of these chimeric constructs, recombinant plasmid constructs, pcDNA3.1-E21a-2A-NS2-31a, pcDNA3.1-E21b-2A-NS2-31b, pcDNA3.1-E22-2A-NS2-32, pcDNA3.1-NS4-51, and pcDNA3.1-NS4-52, encoding the novel antigens, were generated.

**Figure 3:**
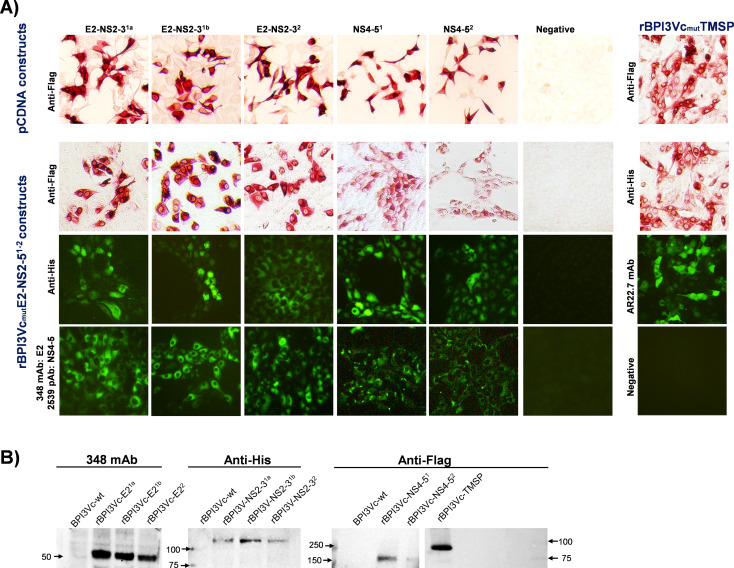
Chimeric BVDV antigens protein expression. **(A)** Top panel: Protein expression by the pcDNA3.1 constructs encoding chimeric BVDV E2-NS2-3^1a,1b and 2^ and NS4–5^1 and 2^ was confirmed by immunocytometric analysis using anti-Flag mAb, monoclonal antibody. Bottom panel: Protein expression by the rescued rBPI3Vc_mut_ E2-NS2-5^1–2^ and rBPI3Vc_mut_TMSP viruses was similarly confirmed as above, but by using: i) Anti-Flag mAb to detect E2^1a,1b,and 2^; ii) Anti-His mAb to detect NS2-3^1a,1b,and 2^; iii) mAb 348 to detect BVDV 1 and 2 E2 glycoprotein; iv) Anti-Flag mAb to detect Flag-tag located at the N-terminal of NS4-5^1,2^ anti-His mAb to detect His-tag located at the C-terminal or anti-BVDV pAb, polyclonal antibody. Expression of TMSP was confirmed by using the following mAbs: anti-Flag, anti-His, and TMSP-specific mAb 22.7. **(B)** Protein expression was also confirmed by Western Blot analysis using mAbs 348, anti-His, and anti-Flag.

**Figure 4:**
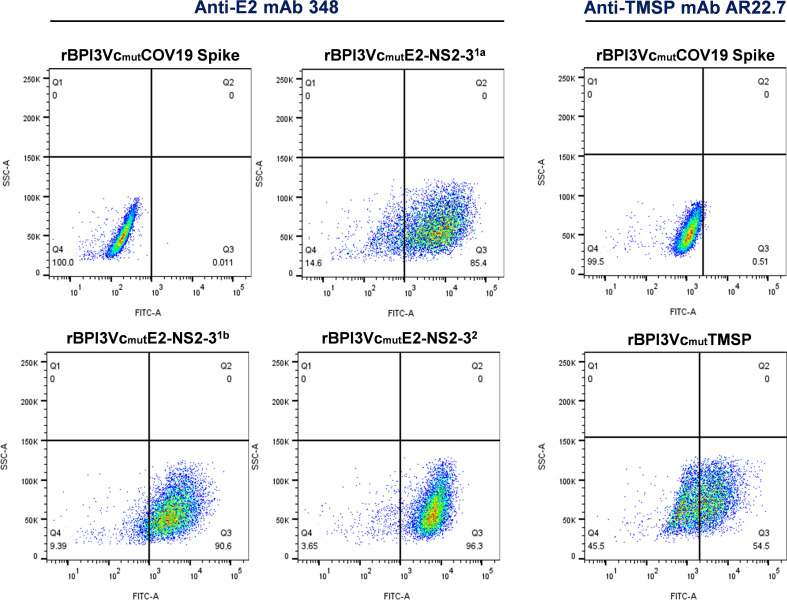
Surface display of chimeric BPI3V E2^1a,1b,2^ antigens. Rescued rBPI3Vc_mut_E2-NS2-3^1a,1b,2^ and rBPI3Vc_mut_TMSP viruses, but not the rBPI3Vc_mut_Spike virus, displayed the E2^1a^, E2^1b^, E2^2^, and the TMSP antigens, respectively, on the surface of infected MDBK cells as confirmed using the E2-specific mAb 348 or the TMSP-specific mAb AR22.7, respectively.

These constructs expressed proteins that were recognized by anti-Flag mAb (**Figure 3A**), anti-His mAb, mAb 348, and pAb 2539 (data not shown). Recombinant viruses generated using the genes encoding the novel BVDV antigens (rBPI3Vc_mut_E2^1a^-NS2-3^1a^, rBPI3Vc_mut_E2^1b^-NS2-3^1b^, rBPI3Vc_mut_E2^2^-NS2-3^2^, rBPI3Vc_mut_NS4-5^1^, and rBPI3Vc_mut_NS4-5^2^), as well as the negative control (rBPI3Vc_mut_TMSP), expressed the encoded antigens as confirmed by immunocytometric analysis, IFA (**Figure 3A**), and Western Blot assay (**Figure 3B**). In addition, flow cytometric analysis showed that the rBPI3Vc_mut_E2^1a^-NS2-3^1a^, rBPI3Vc_mut_E2^1b^-NS2-3^1b^, rBPI3Vc_mut_E2^2^-NS2-3^2^, and rBPI3Vc_mut_TMSP viruses, but not the rBPI3Vc_mut_COVID19 Spike virus, displayed the E2^1a^, E2^1b^, E2^2^, and TMSP antigens, respectively, on the surface of infected MDBK cells (**Figure 4**).

### 3.2 rBPI3Vc_mut_ vectored viruses exhibit genetic stability

**Figure 5:**
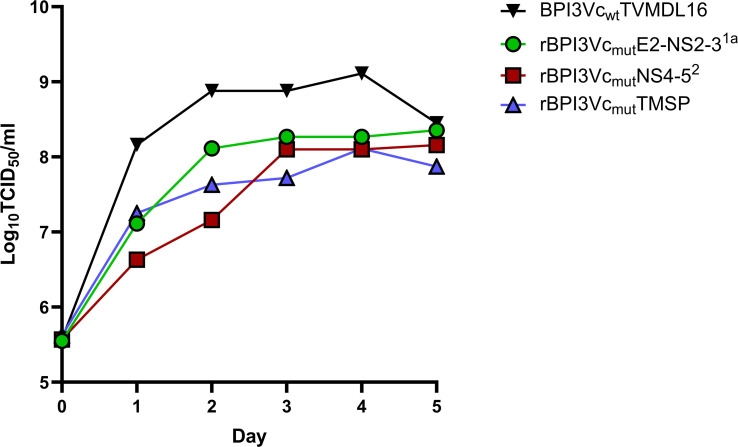
Multicycle replication growth curve. Replication growth curves in MDBK cells infected at an MOI of 1 with either BPI3Vc_wt_TVMDL16 wild-type virus, rBPI3Vc_mut_E2-NS2-3^1a^, rBPI3Vc_mut_NS4-5^2^, or rBPI3Vc_mut_TMSP virus and incubated at 37 °C for four days. Virus aliquots were sampled every 24 hours and quantified by TCID_50_ virus titration.

Replication of the recombinant viruses was not inhibited by the presence of transgenes. Specifically, multicycle replication curves demonstrated that the rBPI3Vc_mut_E2^1a^-NS2-3^1a^, rBPI3Vc_mut_NS4-5^2^, and the rBPI3Vc_mut_TMSP viruses replicated in a similar manner, with titers of 8.35 TCID_50_/ml, 8.16 TCID_50_/ml, and 7.8 TCID_50_/ml recorded on day four, respectively (**Figure 5**). These titers were comparable to the 8.45 TCID_50_/ml titer recorded for the wild-type BPI3Vc TVMDL16 virus on the same day, suggesting that the presence of the ~2.6 kb TMSP or ~5 kb E2-NS2-3^1a^ and NS4–5^2^ transgenes had no effect on overall virus replication (**Figure 5**). Consistent with this preserved replication phenotype, we evaluated the genetic stability of the recombinant vectors during serial passaging.

The vector genome was stable since sequences encoding the N, P, and M genes of the BPI3Vc_mut_ backbone remained identical to those of the original sequence and the initial P2 progenies following passaging the rBPI3Vc_mut_E2-NS2-3^1a^, the rBPI3Vc_mut_E2-NS2-3^1b^, and the rBPI3Vc_mut_NS4–5^2^ viruses in MDBK cells nine times (**Table 3**). For the rBPI3Vc_mut_E2-NS2-3^1a^ virus, nine rounds of passaging resulted in no change to the F and HN genes, an L120 P mutation in the E2^1a^ gene, and a V1030Q mutation in the NS2-3^1a^ gene. Notably, T-to-C point substitutions were observed downstream of the Flag tag of the E2 gene in only the P2 progeny, but not the P9 progeny, which was identical to the original sequence, suggesting that the expanded virus was non-clonal. For the rBPI3Vc_mut_E2-NS2-3^1b^ virus, nine rounds of passaging resulted in no change to the F, HN, and E2-NS2-3^1b^ genes. An N556D mutation was observed in the HN gene of the P2 virus, but not the P9 virus, which was identical to the original sequence. Additionally, mutations in the E2^1b^ gene (Y3H, Y8H, Y10H) and a P689S mutation in the NS2-3^1b^ gene of the P2 virus were carried forward to the P9 progeny. For the rBPI3Vc_mut_NS4–5^2^ virus, nine rounds of passaging resulted in a G324S mutation in the F gene of the P9 virus and some mutations (I25S, I28S, M27T, L39P, V1261A) in the NS4–5^2^ gene. Notably, a G218R mutation in the HN gene of the P2 virus was passed on to the P9 progeny. The L gene remained unchanged across all viruses after the nine passages, except for a consistent T-to-A point substitution in the non-coding region downstream of the stop codon. Overall, the antigenome sequence and the encoded chimeric antigen sequences remained mostly unchanged, demonstrating the genetic stability of the replicating backbone.

**Table 3:** Viral RNA sequence at passage 2 and passage 9.

Virus	Passage	Gene	Identity percentage	Read coverage	Genomic coverage
rBPI3VcmutE2-NS2-3^1a^	2	*N*	100	14446.03787	100
		E2-NS2-3^1a^	98.62804878	1.762729124	7.484725051
		*P*	100	10270.99242	100
		*M*	100	51900.45915	100
		*F_HN*	100	3719.36342	100
		*L*	99.985283296541581	8155.080059	100
	9	*N*	100	963.9187843	99.85052317
		E2-NS2-3^1a^	98.5757884	2.345723014	48.62525458
		*P*	100	397.875	99.33712121
		*M*	100	50133.61768	99.93902439
		*F_HN*	100	178.2311956	99.47215624
		*L*	99.985283296541581	292.5557027	99.85283297
rBPI3VcmutE2-NS2-3^1b^	2	*N*	100	11506.45042	100
		*E2-*E2-NS2-3^1b^	99.88256019	2388.156782	100
		*P*	100	7425.342803	100
		*M*	100	49388.64024	100
		*F_HN*	99.973607812087621	2696.85801	100
		*L*	99.985283296541581	7157.233848	99.9852833
	9	*N*	100	13646.7378	99.93902439
		*E2-*E2-NS2-3^1b^	99.88267501	1418.781953	100
		*P*	100	2606.412556	99.90034878
		*M*	100	940.2556818	99.71590909
		*F_HN*	100	411.7487464	99.94721562
		*L*	99.985283296541581	1044.572627	99.9852833
rBPI3VcmutNS4-5^2^	2	*N*	100	11656.61236	100
		NS4-5^2^	100	3030.389363	100
		*P*	100	8472.04072	100
		*M*	100	2729.286355	100
		*F_HN*	99.973607812087621	53906.25366	100
		*L*	99.985283296541581	5114.187638	99.9852833
	9	*N*	100	16308.4061	99.87804878
		NS4-5^2^	99.13201453	3670.629111	100
		*P*	100	3658.872945	99.95017439
		*M*	100	1454.851326	99.62121212
		*F_HN*	99.947215624175243	948.4283452	99.94721562
		*L*	99.985283296541581	2602.053127	99.9852833

### 3.3 rBPI3Vc_mut_BVDV vaccine is safe and elicits BVDV-specific antibodies

**Figure 6:**
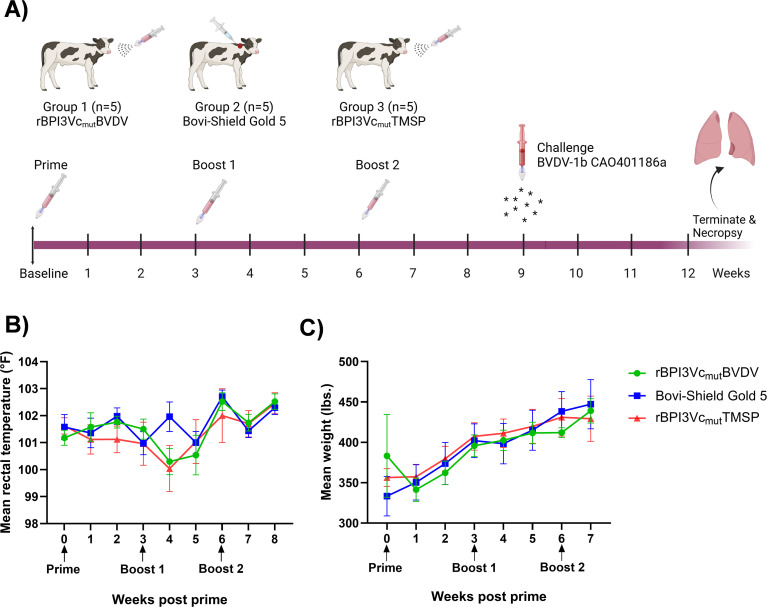
Animal study. **(A)** Immunization design and timeline plan. Calves were assigned to three treatment groups: 1) rBPI3Vc_mut_BVDV vaccine; 2) Bovi-Shield Gold 5 BVDV commercial vaccine; and 3) rBPI3Vc_mut_TMSP control virus. After one week of acclimatization, calves in Groups 1 and 3 received an intranasal prime while calves in Group 2 received a subcutaneous priming dose, followed by booster doses at three-week intervals. Nine weeks post-priming, all calves were challenged via intranasal administration of BVDV Type 1b strain California (CA0401186a). The study was terminated at week 13. Weekly sampling for whole blood, serum and nasal swabs was conducted. **(B)** Mean rectal temperature. **(C)** Mean weight gain for each group measured every week.

Intranasal immunization of calves with the rBPI3Vc_mut_BVDV prototype vaccine, a cocktail of five viruses (rBPI3Vc_mut_E2^1a^-NS2-3^1a^, rBPI3Vc_mut_E2^1b^-NS2-3^1b^, rBPI3Vc_mut_E2^2^-NS2-3^2^, rBPI3Vc_mut_NS4-5^1^, and rBPI3Vc_mut_NS4-5^2^) (Group 1); or with the negative control virus rBPI3Vc_mut_TMSP (Group 3), was well tolerated with no adverse side effects observed following the homologous prime and booster doses (**Table 2**). Similarly, calves immunized subcutaneously with the commercial Bovi-Shield Gold 5 vaccine (Group 2) also exhibited no adverse effects. In all study groups, calves remained healthy throughout the study period, maintained relatively consistent body temperatures, and exhibited steady body weight gain during the immunization phase (**Figures 6A–C**). Having established the safety and tolerability of the immunization regimens, we evaluated the humoral immune responses elicited by the vaccines.

**Figure 7:**
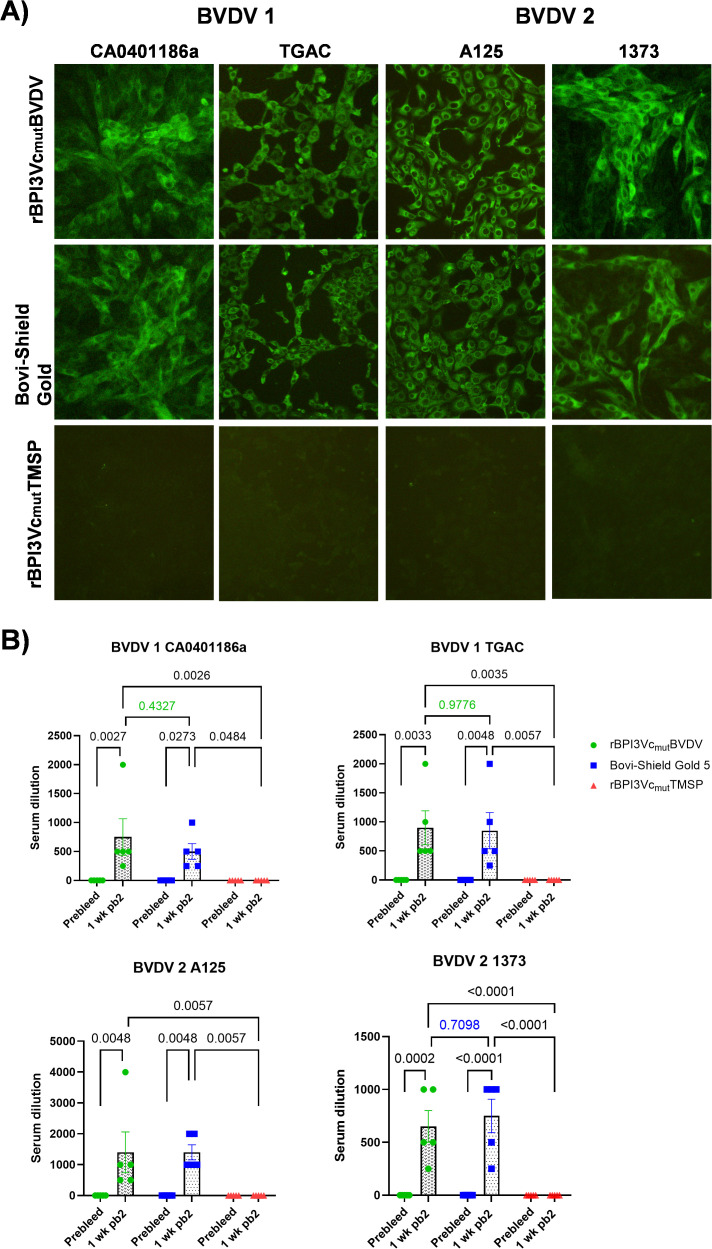
Antibody response. Serum antibodies recognized wild-type BVDV 1 California (CA011401186a), TGAC and BVDV 2 A125, and 1373 viruses as evaluated by Immunofluorescence staining **(A)** and endpoint dilution staining using sera collected before immunization (Prebleed) and one week post-boost 2 (1 wk pb2) **(B)**. Bars represent group means and statistical differences in group means.

Calves immunized with either the rBPI3Vc_mut_BVDV prototype vaccine or the Bovi-Shield Gold 5 vaccine, seroconverted and isotype switched, and the induced BVDV-specific IgG responses recognized wild-type BVDV-1b (CA0401186a and TGAC) and BVDV-2a (A125 and 1373) strains (**Figure 7A**). Compared to the negative controls, both vaccine treatment groups elicited significantly higher BVDV-specific antibody responses against all tested BVDV strains (**Figure 7B**). Notably, calves immunized with the rBPI3Vc_mut_BVDV prototype vaccine elicited stronger, but not significant, mean IgG response against two of the tested viruses (BVDV-1b CA0401186a p=0.4327 and TGAC p=0.9776) compared to the Bovi-Shield Gold 5 MLV vaccinees. While both vaccine treatment groups showed similar mean BVDV-2a A125-specific IgG responses (at 1:1400 serum dilution), one calf in the rBPI3Vc_mut_BVDV treatment group exhibited exceptionally high responses, detected at 1:4000 serum dilution (**Figure 7**).

### 3.4 rBPI3Vc_mut_BVDV prototype vaccine elicited cross-neutralizing antibodies

**Figure 8:**
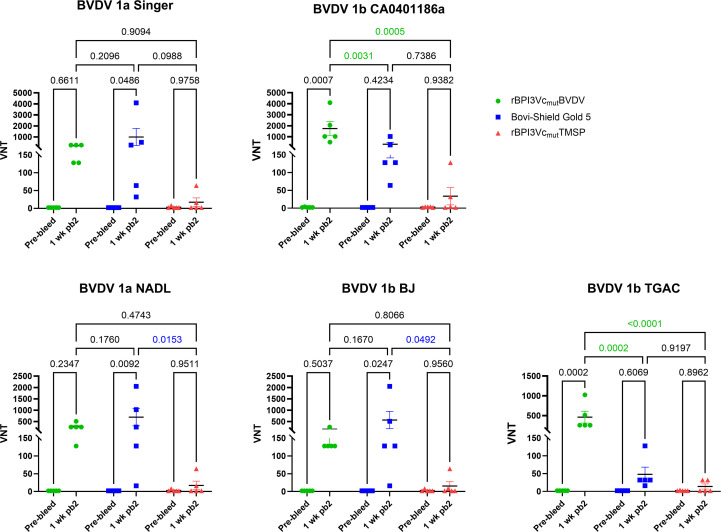
BVDV-1-specific neutralizing antibody titers. BVDV-1-specific neutralization titers in calves immunized with either the rBPI3Vc_mut_BVDV vaccine, Bovi-Shield Gold 5 vaccine, or the rBPI3Vc_mut_TMSP control virus construct were determined by virus neutralization assays against the representative BVDV-1 strains using sera collected before immunization (Pre-bleed) and one-week post-boost-2. Bars represent mean group titers and statistical differences in group means.

Calves immunized with the rBPI3Vc_mut_BVDV prototype vaccine elicited antibodies that neutralized five BVDV-1 and five BVDV-2 strains, as determined by viral neutralizing assays conducted using sera collected one week after the second boost. Notably, the rBPI3Vc_mut_BVDV prototype vaccine elicited significantly higher mean Viral Neutralizing (VN) titers against BVDV-1b CA0401186a and TGAC strains compared to the Bovi-Shield Gold 5 vaccine (p=0.0031 and p=0.0002, respectively) or the negative controls (p=0.0005 and p<0.0001, respectively) (**Figure 8**). Conversely, the Bovi-Shield Gold 5 vaccine elicited higher mean VN titers against BVDV-1a Singer, NADL, and BVDV-1b BJ strains than the rBPI3Vc_mut_BVDV vaccine, with significantly higher titers against NADL (p=0.0153) and BJ (p=0.0492) strains when compared to the negative control treatment (**Figure 8**). Notably, VN titers elicited against BVDV-1 Singer, CA0401186a, NADL, BJ, and TGAC viruses by the rBPI3Vc_mut_BVDV prototype vaccine were 1:128 – 1:256; 1:512 – 1:4096; 1:128 -1:512; 1:128 – 1:256; and 1:256 -1:1024, respectively. On the other hand, VN titers elicited by the Bovi-Shield Gold 5 vaccine against the same BVDV-1 viruses were 1:64 – 1:4096; 1:64 -1:1024; 1:16 – 1:2048; 1:16 – 1:2048; and 1:16 – 1:128; respectively (**Figure 8**). Notably, although the vaccine strains in the commercial Bovi-Shield Gold 5 are BVDV-1a strain NADL and BVDV-2a strain 53637 (53), all calves immunized with the rBPI3Vc_mut_BVDV prototype vaccine had VN titers of more than 1:128 (the protective threshold) against BVDV NADL. In contrast, one calf in the commercial vaccine group had very low titers of 1:16. To further assess the breadth of neutralization, we evaluated responses against BVDV-2a strains.

**Figure 9:**
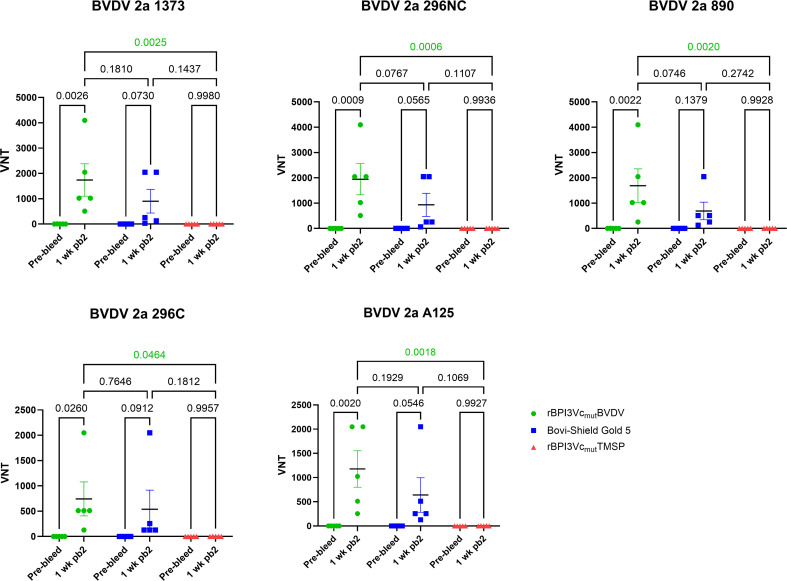
BVDV-2-specific neutralizing antibody titers. BVDV-2-specific neutralization titers in the calves immunized with either the rBPI3Vc_mut_BVDV vaccine, Bovi-Shield Gold 5 vaccine, or the rBPI3Vc_mut_TMSP control virus construct were determined by virus neutralization assays against the representative BVDV-2 strains using sera collected before immunization (Pre-bleed) and one-week post-boost-2. Bars represent mean group titers and statistical differences in group means.

Interestingly, the rBPI3Vc_mut_BVDV prototype vaccine induced significantly higher mean VN titers against all tested BVDV-2a strains 296NC (p=0.0006), 890 (p=0.0020), 296C (p=0.0464), A125 (p=0.0018), and 1373 (p=0.0025) (**Figure 9**). Notably, VN titers elicited against all tested BVDV-2a strains 1373, 296NC, 890, 296C and A125, by the rBPI3Vc_mut_BVDV prototype vaccine were 1: 512 – 1:4096; 1:512 - 1:4096; 1:256 – 1:4096; 1:128 – 1:2048; and 1:256 – 1:2048, respectively. In comparison, titers elicited by the Bovi-Shield Gold 5 vaccine against the same BVDV-2 viruses were 1:32 – 1:2048; 1:64 –1:2048; 1:128 – 1:2048; 1:128 – 1:2048; and 1:128 – 2048, respectively (**Figure 9**).

### 3.5 Post-challenge clinical signs and viremia

**Figure 10:**
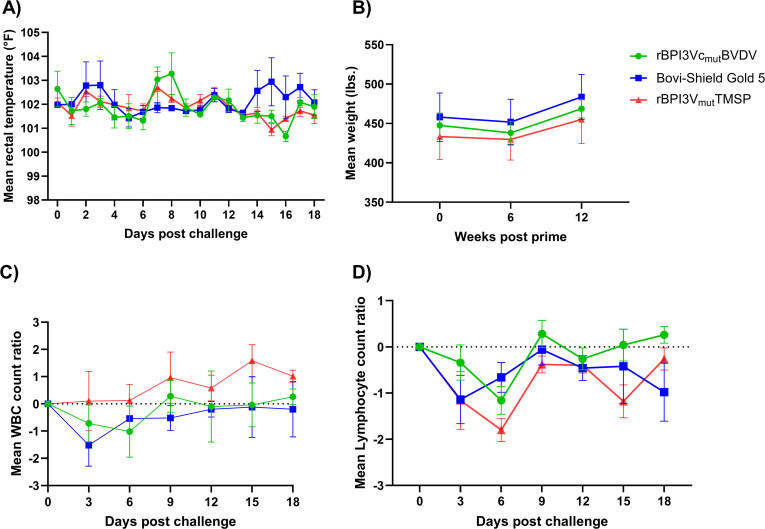
Clinical outcomes post-challenge. **(A)** Mean rectal temperatures recorded daily for each group is plotted; and **(B)** mean gain in body weights recorded on day of challenge, six- and twelve-days post-challenge for each group is plotted. Mean change in **(C)** white blood cell; and **(D)** lymphocyte count ratios for each group quantified on every third day post-challenge is plotted.

Following challenge, mild clinical signs such as decreased appetite, nasal discharge, sluggishness, ocular discharge, and coughing were randomly observed in all treatment groups. All calves experienced an increase in body temperature, with mean peak temperatures recorded on day 7 at 102.7°F in the negative control group, day 8 at 103.3°F in the rBPI3Vc_mut_BVDV treatment group, and day 15 at 102.9°F in the Bovi-Shield Gold 5 treatment group (**Figure 10A**). A slow weight gain was observed in the first week post-challenge in all the treatment groups. By the second week, calves in the rBPI3Vc_mut_BVDV treatment group gained an average of 30.8 lbs., increasing from 438 lbs. to 468.8 lbs. Similarly, those in the Bovi-Shield Gold 5 group gained 32 lbs., increasing from 451.8 lbs. to 483.8 lbs. In contrast, calves in the negative control group had the lowest weight gain, increasing by only 25.2 lbs., from 429.8 lbs. to 455 lbs., although this was not statistically significant (**Figure 10B**). To further characterize these clinical observations, we evaluated hematological changes following challenge.

Interestingly, a decrease in WBC counts was observed in calves immunized with the rBPI3Vc_mut_BVDV (from 7,540/µl on DPC 0 to 6,520/µl on DPC 6) and Bovi-Shield Gold 5 vaccines (from 8,440/µl on DPC 0 to 6,920/µl on DPC 3), but not in the negative control group. The greatest decrease in WBC counts was observed in the Bovi-Shield Gold 5 vaccine group on day 3 post-challenge (**Figure 10C**). This decrease, however, remained within the normal range of 4,700-11,400/µl for healthy calves up to six months old (54). The rBPI3Vc_mut_BVDV vaccinees recovered by day 9 post-challenge, while the Bovi-Shield Gold 5 vaccinees recovered between days 12 and 15 (**Figure 10C**). All treatment groups also exhibited a decrease in Lymphocyte counts, from 4,200/µl on DPC 0 to 3,040/µl on DPC 6 in the rBPI3Vc_mut_BVDV treatment group, from 5,240/µl on DPC 0 to 4,100/µl on DPC 3 in the Bovi-Shield Gold 5 vaccinees, and from 4,720/µl on DPC 0 to 2,920/µl on DPC 6 in the negative control group. However, the negative control group had the lowest mean lymphocyte counts throughout most of the challenge phase (**Figure 10D**). Notably, lymphocyte counts were lower in the Bovi-Shield Gold 5 vaccine group than in the rBPI3Vc_mut_BVDV treatment group for most of the challenge phase. This decrease, however, did not fall below the lower limit of 1,600-1,900/µl for healthy calves of up to six months old (54, 55). By days 15 to 18 post-challenge, only the rBPI3Vc_mut_BVDV vaccine group had fully recovered (**Figure 10D**). Given these differences in clinical and hematological responses, we assessed viral load following challenge.

**Figure 11:**
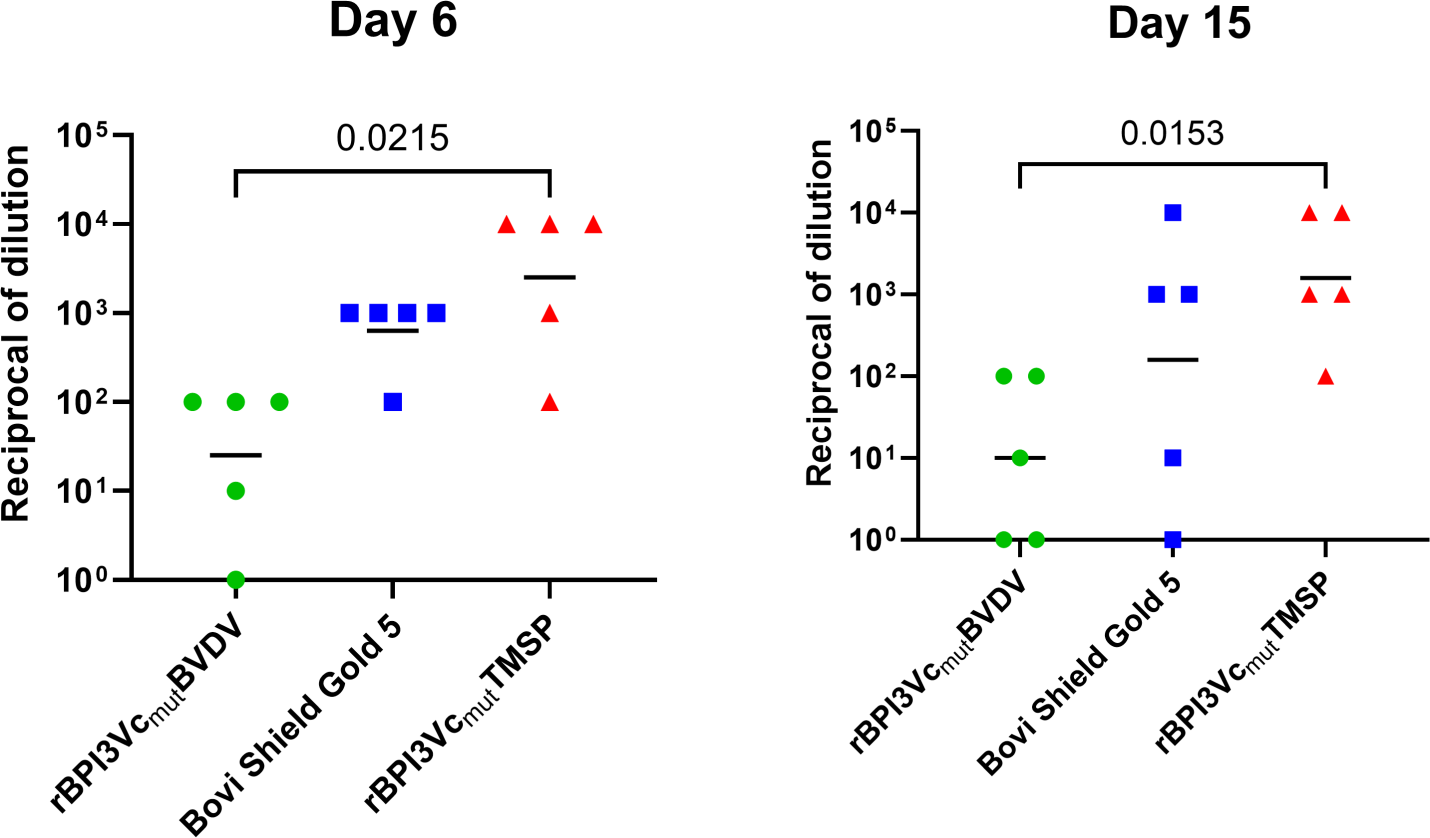
Viremia in calves post-challenge. Viremia was determined in blood samples collected on day 6 **(A)** and day 15 **(B)** post-challenge with BVDV-1b strain CA0401186a. Mean group dilutions are represented by the bars.

Post-challenge, calves immunized with the rBPI3Vc_mut_BVDV prototype vaccine had lower mean BVDV viral load than the Bovi-Shield Gold 5 vaccinees or negative controls. While the BPI3Vc_mut_BVDV vaccinees had lower, but insignificant, mean viremia in blood than the Bovi-Shield Gold 5 vaccinees on day 6 and 15 post-challenge, they showed significantly lower viremia than the negative control group on both day 6 (p=0.0215) and day 15 (p=0.0153) post-challenge (**Figure 11**). Notably, by day 15 post-challenge, the lowest viremia titers were observed in 2 out of 5 calves in the rBPI3Vc_mut_BVDV treatment group and 1 out of 5 calves in the Bovi-Shield Gold 5 vaccinees.

### 3.6 Gross and histopathologic lesions

**Figure 12:**
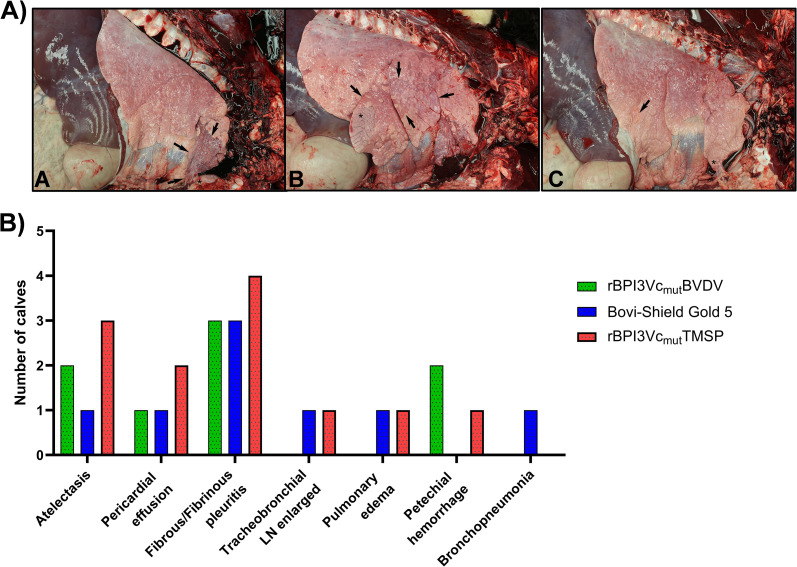
Gross lesions. **(A)** Gross images of lung lesions showing: Panel-**(A)**: Bronchopneumonia and areas of consolidation (arrows) and multiple fibrous adhesions (*); Panel- **(B)**: Atelectasis (arrows) and interlobular emphysema (*); and Panel-**(C)**: fibrinous pleuritis (arrows) and fibrous pleuritis (*). **(B)** Number of calves presenting gross lesions recorded from the thoracic cavity and lung tissues of calves necropsied 18 days post-challenge. Bars represent the number of affected animals for each treatment group.

Pulmonary lesions were observed in all calves but were generally mild and varied among calves (**Figure 12**). Bronchopneumonia was characterized by consolidation of up to 80% of the cranial portion of the right or left cranial lung lobe, along with multiple fibrous adhesions between the visceral pleura of the lung with the parietal pleura and mediastinum (**Figure 12A**(A). Atelectasis was observed in all groups, but was most significant in the cranioventral lungs, affecting up to 70% of the right middle lung lobe, and the caudal and cranial portions of the right cranial lung lobe, with interlobular emphysema present affecting the right middle lung lobe (**Figures 12A, B**). Fibrous or fibrinous pleuritis was also observed in all groups, and was noted along the visceral pleura of the right middle lung lobe and fibrous pleuritis along the visceral pleura of the cranial portion of the right cranial lung lobe (**Figures 12A, C**). Other lesions included pericardial effusion, enlarged tracheobronchial lymph nodes, pulmonary edema, and petechial hemorrhage. Notably, of the seven types of gross lesions observed across all three treatment groups, 4 out of 7 gross lesions were observed in the rBPI3Vc_mut_BVDV vaccinees, and 6 out of 7 lesions were observed in both the Bovi-Shield Gold 5 vaccinees and the negative control rBPI3Vc_mut_TMSP vaccinees. Overall, a total of twelve gross lesions were observed in all the negative control group, whereas in all the vaccinated groups (experimental and commercial vaccinees), only eight gross lesions were reported in total (**Figure 12B**). To further characterize these findings, histopathological evaluation was performed.

**Figure 13:**
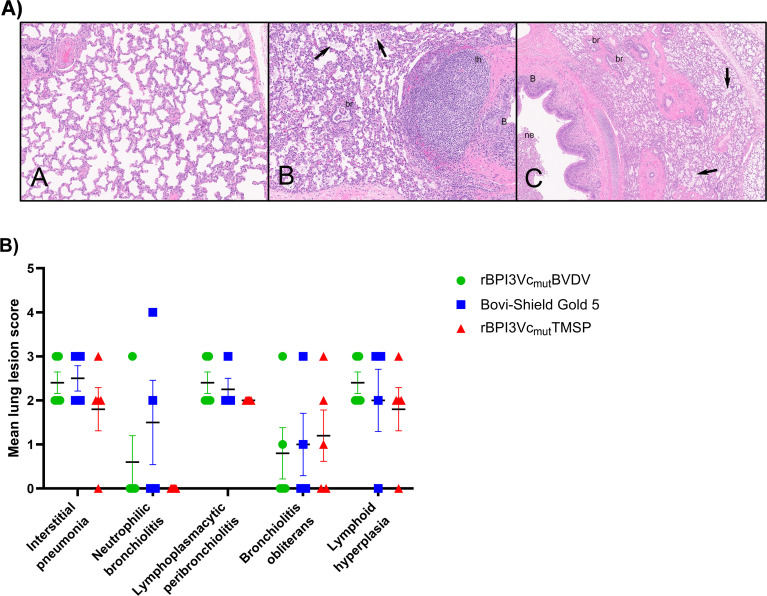
Histopathologic lung lesions. **(A)** Histopathologic sections showing: panel-**(A)** diffuse mild interstitial pneumonia, where the interstitium and alveolar septa are diffusely mildly expanded by increased infiltrating lymphocytes and plasma cells. (10x). panel-**(B)** Diffuse mild to moderate interstitial pneumonia, chronic lymphoplasmacytic bronchitis **(B)** and bronchiolitis, lh, lymphoid hyperplasia of the BALT, bronchiolar associated lymphoid tissue, pulmonary edema (arrows), and early br, bronchiolitis obliterans (10x). Panel- **(C)** Lesions similar to panel-**(B)** along with a ne, neutrophilic exudate within bronchus (B, bronchitis), characteristic of Broncho-interstitial pneumonia at (4x). **B)** Individual lung lesions were scored from sections of the cranial, middle, and caudal lobes of the right and left lung, based on the severity of lesions as follows: (0= normal; 1 = lesion is present; 2 = mild; 3 = moderate; 4 = severe). Individual lung lesion score for each animal is plotted. Bars represent mean lung lesion score for each group.

Most calves developed mild to moderate interstitial pneumonia histologically except one calf in the negative control group (**Figures 13A, B**). Other common histologic lesions were mild to moderate lymphoplasmacytic peribronchiolitis and bronchial or bronchiolar-associated lymphoid tissue hyperplasia. Scoring of the histologic lesions for each group showed no statistically significant difference between the three experimental groups (**Figure 13B**).

## 4 Discussion

Due to antigenic variability, there is a widening gap between current commercial vaccine strains and efficacy against heterologous strains (18, 56, 57). Therefore, there remains a critical need for safer and more effective vaccines for protection against diverse BVDV strains. Current BVDV vaccines in the United States are based on MLVs and Killed virus, which are mainly formulated using representative BVDV-1a and BVDV-2a strains. However, recently identified BVDV-2b and 2c variants in the United States (22), were not neutralized by sera from BVDV-2a MLV vaccinees (18). Additionally, although BVDV-1b is the predominant subspecies in North America (18, 22), nearly all MLVs and KVs are formulated using BVDV-1a. Similar antigenic variation has been observed globally based on viral neutralization tests (24–26) or cell-mediated immune responses (53), highlighting the limitations of current vaccines in providing broad epitope coverage against diverse circulating BVDV strains. To address these limitations, we explored a live-vectored vaccine strategy to develop a contemporary vaccine capable of inducing protection against multiple BVDV strains.

Live-vectored vaccines mimic modified live vaccines in that they replicate *in vivo*, eliciting protective BVDV-specific neutralizing antibodies, CD4^+^ T cell, and CD8^+^ cytotoxic T lymphocyte responses in calves (31). Intranasal live vaccines offer the added advantage of being less susceptible to interference from circulating maternal antibodies, which can otherwise hinder effective immunization in young calves. This study utilized a recently described Bovine parainfluenza-3 virus genotype-C vector (BPI3Vc_mut_), an attenuated virus capable of retaining its infectivity and immunogenicity (45). As such, it elicited neutralizing antibodies against self as well as transgene encoded antigens (45). In the current study, five novel chimeric BVDV antigens, designated E2-NS2-3^1a^, E2-NS2-3^1b^, E2-NS2-3^2^, NS4–5^1^ and NS4-5^2^, were designed based on the consensus of sequenced BVDV antigenic determinants from more than 200 sequences. These antigens were used in generating recombinant BPI3Vc-vectored viruses, which were tested for their potential to confer protection in calves against diverse BVDV strains following intranasal immunization. The rationale for this approach is grounded in the use of consensus-based antigen design to overcome viral diversity.

The generation of chimeric BVDV antigens based on consensus sequences across multiple subspecies represents a strategic approach to address the extensive antigenic diversity characteristic of BVDV. By integrating conserved and variable regions from BVDV-1 and BVDV-2 lineages, this design aimed to broaden epitope coverage and enhance cross-protective potential. The variation observed in sequence conservation among the chimeric constructs, particularly the lower homology of the E2¹^a^ antigen compared with its parental counterparts, underscores the genetic heterogeneity of circulating strains and the importance of including both conserved and strain-specific determinants. Such sequence diversity within the chimeric antigens may facilitate the induction of broadly reactive immune responses capable of recognizing multiple BVDV subspecies. Thus, the consensus-based design framework offers a rational foundation for next-generation vaccines targeting genetically diverse pestiviruses, with the potential to broaden epitope coverage and enhance protective immunity against disparate strains. Previous studies have shown that chimeric antigens can prime broad BVDV-specific antibody repertoire and enhance T cell responses, and confer broader protection against BVDV (39, 50, 58). It has previously been shown that immunization with chimeric antigens can induce broader B cell responses against influenza viruses in mice (59), and confer better protection against Simian HIV-1 in rhesus monkeys (60) as well as against avian influenza in poultry (61). Building on this design strategy, we evaluated the feasibility of expressing these antigens using the BPI3V vector platform.

The successful rescue of recombinant BPI3Vc constructs expressing the chimeric BVDV antigens highlights the feasibility of using this platform to deliver complex, multi-component immunogens. Verification of antigen expression by both immunocytometry and Western blot analysis confirms that the 2A autocleavable motif functioned as intended, enabling efficient and independent expression of the E2 and NS2–3 components within the same viral backbone. This is particularly important for BVDV, where coordinated yet separate presentation of these antigens may enhance the breadth and quality of the immune response. Collectively, these findings underscore the utility of the BPI3V vector system for expressing structurally diverse chimeric proteins and support its further development as a platform for next-generation pestivirus vaccines. In this context, optimizing antigen presentation, particularly for key targets such as E2, is critical for maximizing immunogenicity.

The E2 glycoprotein remains the principal target for BVDV vaccine development because of its essential role in receptor engagement and its rich repertoire of neutralizing and T-cell epitopes (62–64). Although numerous vaccine platforms have focused on E2 alone (65–74), one limitation of traditional subunit approaches is inefficient antigen display in a context that faithfully preserves native conformation. By replacing the native transmembrane and cytoplasmic regions of E2 with those from the BPI3Vc Fusion protein, we sought to enhance surface expression and improve the presentation of both linear and conformational epitopes to B cells. This strategy is particularly relevant for vectored vaccines, where optimal antigen display can strongly influence immunogenicity and breadth of antibody responses (62, 75). Our findings that the recombinant BPI3Vc constructs expressing consensus E2 antigens, but not by a control construct, successfully displayed the E2 ectodomains on the surface of infected cells (**Figure 4**) underscore the effectiveness of this design. These results illustrate how rational modification of antigen anchoring domains can substantially improve epitope accessibility and highlight the broader potential of the BPI3V platform for eliciting robust humoral immunity against diverse BVDV strains. In parallel, the stability of the recombinant vectors is essential for ensuring consistent performance.

The stability of recombinant vectored vaccines is a critical determinant of their suitability for large-scale production and *in vivo* evaluation. Consistent with our earlier findings showing that the BPI3Vc vector platform tolerates inserts of up to ~3.6 kb without compromising viral replication (45), the present study demonstrates that even larger transgenes (~5 kb) can be accommodated with minimal impact on overall viral titers (**Figure 5**). Although these larger inserts produced a somewhat more attenuated growth phenotype during early culture, an outcome consistent with other RNA virus-based vectors (76, 77), this did not compromise the utility of the platform. Importantly, the BPI3Vc backbone and its encoded transgenes exhibited a high degree of genetic stability across nine serial passages, with only a limited number of mutations emerging and no evidence of systematic loss of antigen-encoding sequences. The detection of sporadic mutations in early passage (P2) but not in later passage (P9) populations likely reflects the presence of mixed viral clones early in rescue, underscoring the importance of plaque purification to obtain clonal virus stocks before conducting *in vivo* studies. Overall, these findings reinforce the robustness of the BPI3Vc vector for expressing large, complex antigens and highlight its promise as a platform for vaccine development. Having established vector performance, we evaluated immunogenicity and efficacy *in vivo*.

The comparative immunogenicity and efficacy data highlight important distinctions between the rBPI3Vc_mut_BVDV prototype vaccine and a widely used commercial MLV vaccine, underscoring the potential of the BPI3V vector platform to address limitations of current BVDV control strategies (**Figure 7**). Neutralizing antibodies are a key correlate of protection especially in young calves (27, 78), and the broader and more consistent neutralization profile elicited by the rBPI3Vc_mut_BVDV prototype vaccine suggests enhanced potential for cross-protection against the genetically diverse strains circulating in the United States. The particularly strong responses against BVDV-1b CA0401186a and TGAC, subspecies most frequently implicated in U.S. outbreaks, are noteworthy, as current MLV vaccines often show uneven performance against these variants. The uniformly high titers against BVDV-2 strains further reinforce the value of incorporating consensus-based chimeric antigens into the vaccine design, likely reflecting the greater representation of BVDV-2 sequences in the construct and the inclusion of conserved epitopes across subspecies. Building on these observations, we assessed whether the magnitude of the neutralizing response reached protective thresholds.

Given that VN titers of 1:128 are considered protective against BVDV (18), our findings indicate that the rBPI3Vc_mut_BVDV vaccine elicited protective titers in nearly all calves, outperforming the Bovi-Shield Gold 5 vaccine under the same immunization schedule and conditions. The capacity of the BPI3Vc platform to elicit protective titers in nearly all vaccinated calves, even against strains not explicitly represented in the antigen design (e.g., BVDV-1a NADL), highlights the potential benefits of a respiratory-vectored approach. Intranasal delivery may also confer advantages at the mucosal level, as suggested by recent studies showing enhanced induction of mucosal neutralizing antibodies following intranasal, but not intramuscular, administration of related E2-based vaccines (66). The duration of immunity conferred by the Bovi-Shield Gold 5 vaccine is approximately nine months (79). The duration of immunity provided by the prototype vaccine and the full protective capacity will be empirically established. The current findings position the BPI3V vector as a promising alternative to conventional MLV vaccines, with the potential to improve the breadth and reliability of immunity against evolving BVDV strains given its flexibility to be rapidly updated to match circulating strains. To determine how these immunologic advantages translated into clinical outcomes, post-challenge responses were evaluated.

Acute BVDV infection typically causes different clinical signs such as fever, diarrhea, nasal discharge, coughing, loss of appetite, and ocular discharges along with hematologic changes such as leukopenia and lymphopenia that can lead to immunosuppression (2, 3). Findings from this study indicate that clinical BVDV disease was mild, and no significant differences were observed between the vaccine treatment groups, which was consistent with previous vaccine efficacy studies (29, 30, 78, 80). The observed increase in body temperature toward the end of the study in the calves immunized with the commercial vaccine was due to one calf (ID 5657) that fell sick due to unrelated conditions (**Figure 10A**). Weight gain affects the economic impact of beef animals. During the challenge phase, vaccinated calves exhibited higher weight gain compared to the non-vaccinated controls (**Figure 10B**), similar to other challenge studies showing significantly higher carcass weight in vaccinated animals (78, 81). Interestingly, all immunized calves exhibited a decrease in white blood cell count, while the non-vaccinated negative control calves did not. Although all treatment groups exhibited a decrease in lymphocyte counts, the greatest decrease was observed in the negative control group, followed by the Bovi-Shield Gold 5 vaccine group (**Figures 10C, D**). Importantly, the observed decrease in this study were within the normal range of 4,700-11,400/µl (for WBCs) and not lower than 1,600-1,900/µl lymphocyte counts, in healthy calves of up to six months old (54, 55). A recent study reported that calves immunized intranasally, but not parenterally, with a BVDV MLV vaccine in the face of maternal antibodies, developed leukopenia upon challenge (82, 83). However, we observed decreased WBC counts in both the intranasally immunized rBPI3Vc_mut_BVDV and parenterally immunized Bovi-Shield Gold 5 groups. It is possible that administering three immunization doses, three weeks apart (despite Bovi-Shield Gold 5 being recommended for re-vaccination at least after nine months or annually (79), may have induced a stronger inflammatory response upon challenge. This may have led to a cytokine surge and lymphocyte margination causing leukocytes to move from blood stream into tissues, leading to transient decrease in WBC counts (84, 85), as supported by data showing the presence of more lymphocytes in the vaccinated groups than the negative control group (**Figure 10D**). Consistent with these clinical and hematologic observations, virologic outcomes following challenge were examined.

The reduced viremia observed in calves immunized with the rBPI3Vc_mut_BVDV prototype vaccine underscores the functional relevance of the stronger neutralizing antibody responses elicited by this platform. Calves receiving the prototype vaccine showed markedly lower viral loads following challenge compared with both the commercial Bovi-Shield Gold 5 vaccine and unvaccinated controls, aligning with the well-established association between high VN titers and effective control of BVDV replication (**Figure 11**). These findings are consistent with numerous reports demonstrating that vaccines capable of inducing robust humoral immunity substantially limit viremia after exposure (30, 39, 50, 73, 74). To further evaluate the impact of vaccination on disease pathology, gross and histologic lesions were assessed.

Gross and histologic lesions observed across groups largely paralleled those described in prior BVDV vaccine studies, providing confidence in the biological relevance of the challenge model (68, 69, 82, 86). Importantly, animals immunized with the rBPI3Vc_mut_BVDV vaccine exhibited fewer categories of gross lesions than either commercial vaccine recipients or negative controls, suggesting a trend toward improved clinical protection. For example, bronchopneumonia, recognized as a hallmark lesion of BVDV infection (69, 87), was detected only in a calf immunized with the commercial vaccine and not in those receiving the prototype vector. Although histologic abnormalities were present in all groups, the interpretation of lesion severity remains complex, as factors such as antigen dose and the repeated immunization schedule may influence pulmonary immune responses. These observations support the potential of the rBPI3Vc_mut_BVDV platform not only to enhance neutralizing antibody breadth but also to mitigate virologic and pathological outcomes following infection. Future studies examining optimized dosing strategies and long-term immunity will help clarify the full protective capacity of this vectored vaccine approach. Taken together, these findings inform the broader implications of this vaccine platform.

Collectively, our findings highlight the promise of the BPI3Vc vector as a platform for developing broadly protective BVDV vaccines. Because neutralizing antibodies are critical for preventing initial infection, particularly in neonates and yearlings exposed to persistently infected animals (35, 42), vaccine strategies capable of eliciting high and cross-reactive antibody titers remain essential for effective control. Although several live-attenuated and subunit vaccines have been evaluated for fetal, feedlot, and herd-level protection, most rely on single-strain antigens and therefore provide limited coverage against the extensive antigenic diversity of circulating BVDV strains (29, 30, 66, 67, 73, 74, 81, 86, 88–90). This challenge is underscored by recent studies demonstrating poor cross-neutralization of field isolates by commercial vaccines formulated with BVDV-1a or other individual strains (25, 26, 71). In contrast, the consensus-based chimeric antigens used in our prototype vaccine were derived from more than 200 BVDV genomes spanning multiple subspecies, enabling the incorporation of both conserved and variable epitopes representative of global diversity. This design translated into broad neutralization capacity, particularly against BVDV-2 strains and the predominant BVDV-1b lineage circulating in the United States, subspecies for which cross-protection has historically been inconsistent. Although neutralization against BVDV-2b and -2c strains was not directly assessed, their inclusion in the antigen design supports the expectation of comparable protection. Collectively, these findings support advancing next-generation vaccine design and field validation of efficacy and durability of immunity.

In summary, this work demonstrates the feasibility and promise of using consensus-based chimeric E2-NS2–3 and NS4–5 antigens to address the antigenic diversity that has long challenged effective BVDV vaccine development. The ability of the BPI3Vc vector to display E2 antigens on the surface of infected cells is particularly noteworthy, as this feature enables efficient B-cell engagement and likely contributes to the breadth of the neutralizing antibody responses observed. Intranasal delivery of the recombinant virus further underscores the potential of a respiratory-targeted vectored vaccine to elicit strong mucosal and systemic immunity capable of reducing viremia following challenge with wild-type BVDV. These findings support a broader conceptual framework in which rationally engineered, sequence-informed antigen design, combined with a flexible and easily upgradable vaccine, offers a powerful strategy for overcoming BVDV antigenic variability. While the present pilot study provides an encouraging proof-of-concept data, several refinements will be essential to advance this platform toward practical application. Future work should include optimizing dosing and immunization schedules, particularly to determine whether a prime-boost or prime-boost-boost regimen yields the most durable protection; evaluating neutralization breadth against additional BVDV-2 subspecies such as 2b and 2c, which were incorporated into the antigen design; and characterizing the T-cell response, given the intentional inclusion of defined T-cell epitopes within the chimeric constructs.

## Data Availability

The data presented in the study are deposited in the Dryad repository: DOI: 10.5061/dryad.83bk3jb7t.
